# Plant immune receptors mimic pathogen virulence targets

**DOI:** 10.18632/oncotarget.4838

**Published:** 2015-06-15

**Authors:** Panagiotis F. Sarris, Jonathan D.G. Jones

**Affiliations:** The Sainsbury Laboratory, Norwich Research Park, Norwich, UK

**Keywords:** Chromosome Section, WRKY, effectors, hypersensitive response, cell death

All multicellular eukaryotes are susceptible to pathogens. Animal hosts have developed cell-autonomous innate immunity, and chordates have additionally evolved an adaptive immune system to defend against pathogen invasion. In contrast, plant immunity is entirely innate, and relies on the ability of individual cells to activate defense upon pathogen detection by either cell surface or cytoplasmic immune receptors [1].

Microbial pathogens of eukaryotes use similar strategies to infect animals, invertebrates and plants. In order to colonize their host, microbes have developed sophisticated protein secretion systems for the delivery of virulence effector proteins into the host cell cytoplasm. The effector proteins modulate host immune responses and promote pathogenesis.

The plant innate immunity system relies on membrane and cytoplasmic immune receptors. The plasma membrane-embedded PRRs (Pattern Recognition Receptors) have evolved to detect microbial PAMPs (Pathogen Associated Molecular Patterns) and activate PAMP- (or Pattern-) Triggered Immunity (PTI). The intracellular immune receptor genes encode NB-LRR (Nucleotide Binding-Leucine Rich Repeat), which structurally and functionally resemble mammalian Nod-Like Receptors (NLRs). The NB-LRR receptors accomplish intracellular detection of pathogen effectors, either directly or indirectly, and activate an immune signalling pathway (Effector Triggered Immunity, or ETI) that often culminates in a Hypersensitive cell-death Response (HR) [1]. The importance of NB-LRRs to plant defense is illustrated by the expanded complement of NB-LRRs in plants compared to NLRs in mammals. For example, in *Arabidopsis thaliana* ~120 full length NB-LRRs have been identified, while most mammals have ~20 NLRs [2]. Like animal NLRs, plant immune receptors have a modular structure and can work in pairs, both of which are required for defense activation upon recognition of specific pathogen effectors. However, how such intracellular immune receptor complexes activate defense solely upon recognition of microbial molecules is poorly understood.

An NB-LRR pair is required in *Arabidopsis* for the detection of two unrelated bacterial pathogen effectors, PopP2 (of the root pathogen *Ralstonia solanacearum*) and AvrRps4 (of the leaf pathogen *Pseudomonas syringae* pv. *pisi*). The pair consists of the NB-LRR proteins RPS4 (Resistance to *Pseudomonas syringae* 4) and RRS1-R (Resistance to *Ralstonia solanacearum* 1). RRS1-R is an atypical NB-LRR, since it also contains a C-terminal WRKY DNA-binding motif. It is noteworthy that the plant WRKY proteins are sequence-specific DNA-binding transcription factors (TFs) with important roles in immune responses; mutations in several *Arabidopsis* WRKY TF genes are associated with reduced pathogen resistance [1]. In the absence of an appropriate elicitor, RRS1-R acts as a negative regulator of the complex, keeping the RPS4 protein in an “off” state [6]. An RRS1-R mutant allele that contains a leucine insertion in its WRKY-domain (first reported as “sensitive to low humidity-1” (*slh1*)) cannot bind DNA *in vitro,* is autoactive and confers constitutive activation of defense [4].

**Figure 1:**
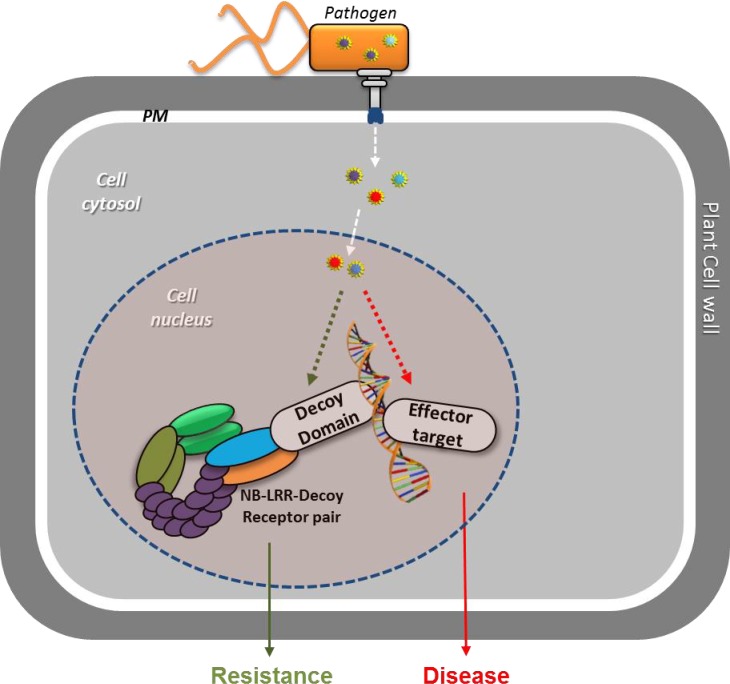
In susceptible plants, pathogens deliver effector proteins to interfere with plant defenses, so that they can colonize the host tissues and cause disease Resistant plants have receptors with an incorporated “Decoy” domain that mimics effector targets. The decoy domain enables plants to detect effectors that specifically target this domain and activates defenses to contain the pathogen and halt the infection. PM: cell plasma membrane.

Two recent papers in Cell [3; 5], reveal the role of the incorporated C-terminal WRKY-domain of RRS1-R as a decoy for the detection of pathogen effectors that interfere with plant WRKY transcription factors (Figure 1). Both PopP2 and AvrRps4 effectors specifically interact with the RRS1-R WRKY domain. PopP2 is an acetyltransferase of the YopJ family. Acetylation of a specific lysine of the RRS1-R WRKY domain (K1221) disrupts DNA binding, leads to defense activation and cell death induction. Likewise, several *Arabidopsis* WRKY TFs are acetylated by PopP2. PopP2 acetylation of WRKY TFs results in reduced DNA binding and leads to compromised induction of basal immune responses. AvrRps4 also interacts with a number of different WRKY TFs. However, Sarris et al. (2015) showed that the interaction of AvrRps4 with WRKY TFs or the RRS1-R WRKY-domain does not lead to compromised DNA binding, which might indicate a mechanistic difference between PopP2 and AvrRps4 defense activation.

These findings provide a simple and unifying concept for how a plant immune receptor complex recognizes two unrelated bacterial effector proteins. PopP2 and AvrRps4 are virulence factors, which have evolved to manipulate host immunity by specific targeting of WRKY TFs; the WRKY-domain of RRS1-R has co-evolved as an “Integrated Decoy” enabling plants to detect effectors whose function is to interfere with defense mechanisms mediated by WRKY TFs [7].

An in-depth interrogation of sequenced plant genomes revealed the presence of other incorporated protein domains in plant NB-LRRs (Sarris PF *unpublished data*). Our analysis revealed many other single and paired NB-LRRs containing additional domains of known and unknown function. It is likely that these domains reflect effector targets in host plants. This enables discovery of new genes that are likely to have an effect on host colonization by either plant innate immunity components or be more general pathogen susceptibility targets.
